# On the Uses of Neutral Carbonate of Magnesia in Calculous and Other Complaints

**Published:** 1815-12

**Authors:** Anthony Meyler


*For the London Medical and Physical Journal.*

### *On the Uses of Neutral Carbonate of Magnesia in Calculous and other Complaints.* By Anthony Meyler, M.D.

THE benefits which the physician has derived from the researches of the chemist, have, perhaps, in few cases been more fully manifested than in the diseases of the urinary organs. At no very distant period, all our knowledge and experience of the cause and treatment of calculi was purely empirical, the treatment was indicated by no accurate or legitimate views, and the same remedy proposed for calculi originating from opposite causes. To the philosophic and enlightened researches of modern chemists, we are indebted for a great mass of useful knowledge—the different species of urinary calculi have been accurately analysed, their composition has been ascertained, the mode of distinguishing each species has been well defined, analogies tending to enlighten our researches in athritic and other diseases have been pointed out, while the theoretic views originating from these discoveries have led to highly important practical results.

Though the combined researches of the physiologist and chemist have not yet been able to discover an actual solvent for the stone already formed in the body, they have in many cases been able to check its enlargement, so that it may be voided by the bladder, and in a great variety of cases have altogether prevented its formation. The analysis of Dr. Pearson, of Fourcroy, and of Vauquelin, had ascertained that the greater number of urinary calculi consist of uric acid. Mr. Brande has ascertained that uric acid is almost the sole constituent of many calculi, and forms the nucleus of by far the greater number. Dr. Wollaston, with peculiar accuracy and ingenuity, has ascertained the composition of a variety of calculi, whose constituent parts were previously unknown, and has rendered the most essential services to this branch of medicine, by ascertaining that gouty concretions consist of uric acid and soda; thereby enabling us to generalize our doctrines and confirming the analogy (which before his discoveries was only supposed to exist) between athritic and calculous complaints.

The inquiries of Sir Everard Home into the functions of the stomach, led Mr. Brande to conclude that the greater number of calculous complaints would be altogether prevented by, in the first instance, introducing such substances into the stomach as would prevent the generation of acidity there and in the *primæ viæ*, the generally supposed cause of the morbid increased secretion of uric acid, and of the greater number of calculi of that kind. Mr. Hatchett suggested, for this purpose, magnesia, and experience has proved that this earth is not only best calculated to neutralize and carry off the acid, but its constant exhibition is also far less injurious to the digestive organs than any of those other remedies that had been previously employed. It has accordingly got into general use, and there are few calculous or gouty patients that do not occasionally resort to this valuable remedy. Like most other medicines, it has been frequently improperly exhibited, and has even served to encrease the complaint it was intended to relieve. It not only tends to aggravate some calculous complaints, but, when continued for too long a time after the symptoms, connected with uric acid, have been removed, instead of depositing red sand, the urine acquires from its exhibition a tendency to deposit white sand, which Dr. Wollaston ascertained to consist of an ammonia-co-magnesian phosphate, with sometimes phosphate of lime. When mineral acids are given, this deposit of white sand is prevented, but they induce the return of the red; vegetable acids do so in a less degree than the mineral ones; but carbonic acid is found peculiarly useful, it not only contributes to prevent the morbid irritability of the stomach and bladder, but enables the urine to retain the phosphates in solution, the phosphates under its exhibition being voided in solution; but, when the carbonic acid is discontinued, they are again deposited in the form of a white sand. Independently of these considerations, the exhibition of magnesia is in other instances attended with injurious consequences; where no acid exists in the stomach, it remains in an uncombined state in the stomach and bowels, and in some instances has been ascertained to injure materially the digestive organs; nor can we, in any instance, ascertain the quantity of magnesia nesia necessary to neutralize whatever acid may be in the stomach; it may also form a nucleus for calculous concretions or increase those already formed.

The exhibition of the neutral carbonate of magnesia may, perhaps, be found to answer all the purposes for which this earth is given, without any of those injurious consequences which the improper exhibition of magnesia produces. Having read in some of the late journals, that Mr. Richards, of Bath, had succeeded in forming a solution of magnesia in water, impregnated with fixed air under strong pressure, but where no mention is made of the method used or the quantity of magnesia that was dissolved; I instituted a variety of experiments to ascertain what quantity of magnesia might be dissolved in this manner, with the view of administering it as a purgative; and, by the means of the powerful soda-water apparatus of Mr. Laurence, I have succeeded in forming a transparent solution, in the proportion of fifteen grains, of the sub-carbonate of magnesia of commerce to the ounce of water. Mr. Laurence has, under my direction, made up this solution in bottles containing seven ounces, and in this proportion it forms a purgative equally active with an ounce of Epsom salts. The solution is also highly impregnated with carbonic acid, and is divested of much of that disagreeable taste which saline purgatives possess, and which unfortunately frequently limits their application. Here then we are in possession of a neutral salt equally efficacious with any other we possess, and which, from the loose state of combination with which the carbonic acid and the magnesia are held together, is easily decomposed by any acid it may meet with in the *primæ viæ*. It, perhaps, will be found not less powerful than magnesia itself in preventing calculous and athritic concretions, without any of the injurious consequences which the injudicious exhibition of magnesia frequently produces. In scrofula, rachitis, dyspepsia, and other complaints attended with, or originating from, an acid in the digestive organs, it may be found to be the best purgative we can exhibit; its decomposition by any acid it meets with in the stomach or bowels, must necessarily be gradual; and it is unnecessary to mention to chemists the great advantages of presenting bodies to each other in their nascent state, which will be accomplished by this salt immediately neutralizing any acid that may be formed through the whole extent of the alimentary canal. It has been proposed to substitute carbonated magnesia water for soda water; and, in those cases where purgatives are not indicated, it may with safety and advantage supersede that very agreeable beverage. In the proportion of ten or twenty grains of magnesia to half a pint a pint of water, super-saturated with fixed air, it is equally as agreeable to the palate as soda water; and its constant exhibition would contribute to mitigate, and perhaps obviate, many diseases. Dr. Egan, an eminent physician in the city, who published a very able paper on athritic and calculous Complaints, in the Transactions of the Royal Irish Academy, had several years hence succeeded in procuring crystals of the carbonate of magnesia in a Nooth's apparatus; but such means are too limited for general use, and the production of the neutral carbonate of magnesia in this manner must necessarily have been in very small quantities.

I am at present engaged in a series of experiments to ascertain more fully the influence of this medicine in calculous and other complaints, and to determine with more accuracy than I have yet been able to do the proportions of its constituent parts. The following is the analysis I have made of the preparation made by Mr. Laurence; but the analysis was performed under circumstances that did not admit of strict accuracy, my views being as yet more directed to it as a medicine than to ascertain accurately its chemical relation.

1. Each bottle of the water contains seven ounces by measure, in which a great quantity of carbonic acid was condensed.

2. The excess of the carbonic acid holding the magnesia in solution is expelled by the application of a moderate heat, and common sub-carbonate of magnesia deposited.

3. One ounce of the water evaporated to dryness yielded fifteen grains of common sub-carbonate of magnesia.

4. Ten grains of this magnesia, obtained by evaporation and heat, were treated with diluted sulphuric acid, 3·5 grains of carbonic acid were obtained, or 35 per cent. nearly agreeing with Kirwan's estimation of the magnesia of the shops, viz. containing

| | |
|---|---|
| Magnesia ---- | 45 |
| Carbonic acid - | 34 |
| Water ------ | 21 |
| | 100 |

5. As the application of heat prevented my obtaining the carbonate of magnesia in a crystallized form, recourse was had to spontaneous evaporation; and a portion of the water, exposed on a dish to the air, had its surface speedily covered with a film, resembling the crust on lime water similarly treated; a mass of apparently minute crystals was likewise deposited, which were collected dried on bibulous paper, and submitted to the following experiments:—

1. Ten

1. Ten grains were treated with diluted sulphuric over mercury, six cubic inches of carbonic acid were obtained, which would weigh 2·82 grains, as 100 cubic inches of carbonic acid weigh 47 grains; from this experiment we learn that the salt contains 2[illegible]·2 per cent. of carbonic acid.

2. Ten grains of the salt were heated red for half an hour in a platina crucible, to drive off the carbonic acid and water; the pure magnesia left behind weighed 2·5 grains, or 25 per cent. consequently the composition of the salt must be

| | |
|---|---|
| Magnesia | 25· |
| Carbonic Acid | 28·2 |
| Water | 46·8 |
| | 100 |

Now, according to Fourcroy's experiments (*Annal. de Chimie*, tom. ii. p. 278,) crystallized carbonate of magnesia, obtained by mixing together a solution of the carbonate of soda and sulphat of magnesia, is composed of

| | | |
|---|---|---|
| Magnesia | 25 | same as Kirwan's crystals. |
| Carbonic acid | 50 | |
| Water | 25 | |
| | 100 | |

Fourcroy also mentions a common carbonate of magnesia, which agrees tolerably well with the salt I have procured, as to the proportion of magnesia and carbonic acid; this he states contains

| | |
|---|---|
| Magnesia | 40 |
| Carbonic acid | 48 |
| Water | 12 |
| | 100 |

But the proportion of water in the salt I have procured, so much exceeds the quantity mentioned by any chemist, in the composition of carbonate of magnesia fully crystallized, that one is led to consider the fact as a new observation. Probably the salt may be the common carbonate of magnesia, with an extraordinary quantity of water combined, i. e. a super-hydrocarbonated magnesia.

33, *Lower Gardner-street, Dublin;*
*October* 4, 1815.

